# Duração do Sono, Genética e Aterosclerose: Desafios e Oportunidades

**DOI:** 10.36660/abc.20240593

**Published:** 2024-10-22

**Authors:** Ana Vitoria Vitoreti Martins, Larissa T. Krul, Luciano F. Drager

**Affiliations:** 1 Hospital das Clínicas da Faculdade de Medicina da USP Instituto do Coração Unidade de Hipertensão São Paulo SP Brasil Unidade de Hipertensão - Instituto do Coração (InCor), Hospital das Clínicas da Faculdade de Medicina da USP, São Paulo, SP – Brasil

**Keywords:** Sono, Aterosclerose, Genética, Doença Cardiovascular

A aterosclerose, o mecanismo unificador para a maioria dos eventos de infarto do miocárdio e acidente vascular cerebral fatais e não fatais, é um processo multifatorial que vem aumentando em prevalência em todo o mundo. Os fatores de risco tradicionais para aterosclerose foram extensivamente estudados, mas evidências recentes apontaram que eles explicavam aproximadamente apenas 40-50% da variabilidade dos marcadores de dano arterial e aterosclerose.^1^ Portanto, um interesse crescente tem sido dedicado à exploração do papel relativo dos fatores de risco "não tradicionais". Um dos candidatos promissores são os distúrbios do sono. De fato, a relação entre sono e saúde cardiovascular tem sido cada vez mais explorada na literatura.^2,3^ Embora a maioria das evidências se baseie no impacto da apneia obstrutiva do sono (AOS) na aterosclerose,^4^ os potenciais efeitos aterogênicos de extremos de duração do sono foram investigados recentemente,^5,6^ considerando sua associação positiva com desfechos cardiovasculares ruins. Curiosamente, evidências anteriores mostraram consistentemente que não apenas a curta, mas a longa duração do sono (geralmente definida como >8 ou >9hs) aumentou o risco para esses eventos, confirmando uma associação em forma de U entre a duração do sono e os desfechos cardiovasculares.^7^ Essas evidências anteriores abrem caminho para explorar mecanismos potenciais pelos quais os extremos de duração do sono podem aumentar o risco cardiovascular.

Nesta edição dos Arquivos Brasileiros de Cardiologia, Xu et al. ^8^ avaliaram a associação entre a duração do sono e o risco de aterosclerose usando uma abordagem de randomização mendeliana (RM) com dados de estudos de associação genômica ampla (GWAS). Duas grandes coortes da Europa foram incluídas para análise. Os principais resultados indicaram que, ao contrário da hipótese inicial, não há associação causal significativa entre a duração do sono e a aterosclerose. O estudo concluiu que a duração do sono geneticamente prevista não afeta o risco de aterosclerose e sugeriu mais pesquisas para explorar essa relação em maiores detalhes. Os pontos fortes do estudo incluem o grande tamanho da amostra e o uso de um estudo de RM. Essa estratégia permite evitar vieses comuns observados em estudos observacionais. No entanto, limitações importantes merecem comentários apropriados que impeçam conclusões definitivas nesta importante área de pesquisa: 1) uma questão subjetiva definiu a duração do sono. Não é incomum encontrar percepções errôneas sobre a duração real do sono;^9^ 2) o polimorfismo de nucleotídeo único (SNPs) escolhido explica apenas parcialmente a variabilidade da duração subjetiva do sono nesta investigação. Este fato pode contribuir para menor poder estatístico na identificação de situações com correlações fracas; 3) a ‘aterosclerose’ foi identificada usando a Classificação Internacional de Doenças (CID) em vez de medidas objetivas. Há certamente uma subnotificação da carga real da aterosclerose nesta população; 4) outros distúrbios do sono (destacando o papel da AOS) são consistentemente associados a marcadores prevalentes e incidentes de aterosclerose,^10–12^ mas não foram estudados na presente investigação; 5) a aplicabilidade dos resultados a populações não europeias merece investigações adicionais.

Concluindo, o estudo conduzido por Xu et al.^8^ lança luz sobre a complexidade do sono e da aterosclerose em termos de caracterização apropriada e como estimar o risco de distúrbios do sono considerando a genética e outros fatores de risco tradicionais para aterosclerose. Como um potencial fator de risco cardiovascular, a pesquisa do sono ainda é uma "novidade no pedaço" em Cardiologia, merecendo investigações mais aprofundadas e apropriadas.^12^
