# Supplementary material for: Duração do Sono, Genética e Aterosclerose: Desafios e Oportunidades
Source: Arq Bras Cardiol. 2024 Oct 22;121(9):e20240593. [Article in Portuguese] doi: 10.36660/abc.20240593 (PMC11634226; doi:10.36660/abc.20240593)
Supplement: Translation

# Translation: Sleep Duration, Genetics, and Atherosclerosis: Challenges and Opportunities

**DOI:** 10.36660/abc.20240593i

**Authors:** Ana Vitoria Vitoreti Martins, Larissa T. Krul, Luciano F. Drager

**Affiliations:** 1 São Paulo SP Brazil Unidade de Hipertensão - Instituto do Coração (InCor), Hospital das Clínicas da Faculdade de Medicina da USP, São Paulo, SP – Brazil

**Keywords:** Sleep, Atherosclerosis, Genetics, Cardiovascular Disease

Atherosclerosis, the unifying mechanism for most of the non-fatal and fatal myocardial infarction and stroke events, is a multifactorial process that has been increasing in prevalence worldwide. Traditional risk factors for atherosclerosis have been extensively studied, but recent evidence pointed out that they explained approximately only 40-50% of the variability of markers of arterial damage and atherosclerosis.^1^ Therefore, growing interest has been devoted to exploring the relative role of the "nontraditional’ risk factors. One of the promising candidates is sleep disorders. Indeed, the relationship between sleep and cardiovascular health has been increasingly explored in the literature.^2,3^ While most of the evidence relies on the impact of obstructive sleep apnea (OSA) on atherosclerosis,^4^ the potential atherogenic effects of extremes of sleep duration has been recently investigated,^5,6^ considering its positive association with poor cardiovascular outcomes. Interestingly, previous evidence consistently showed that not only short but long sleep duration (usually defined as >8 or >9hs) increased the risk for these events, confirming a U-shaped association between sleep duration and cardiovascular outcomes.^7^ This previous evidence paves the way for exploring potential mechanisms by which sleep duration extremes may increase cardiovascular risk.

In this edition of the *Arquivos Brasileiros de Cardiologia*, Xu et al.^8^ evaluated the association between sleep duration and the risk of atherosclerosis using a Mendelian randomization (MR) approach with data from genome-wide association studies (GWAS). Two large cohorts from Europe were included for analysis. The main results indicated that contrary to the initial hypothesis, there is no significant causal association between sleep duration and atherosclerosis. The study concluded that genetically predicted sleep duration does not affect the risk of atherosclerosis and suggested further research to explore this relationship in greater detail. Strengths of the study include the large sample size and the use of a MR study. This strategy allows for avoiding common biases observed in observational studies. However, important limitations deserve appropriate comments precluding definitive conclusions in this important research area: 1) a subjective question defined sleep duration. It is not uncommon to find misperceptions about the real sleep duration;^9^ 2) the chosen Single Nucleotide Polymorphism (SNPs) only partially explain the variability of subjective sleep duration in this investigation. This fact may contribute to lower statistical power in identifying situations with poor correlations; 3) ‘atherosclerosis’ was identified by using the International Classification of Diseases (ICD) rather than objective measurements. There is certainly an underreport of the real burden of atherosclerosis in this population; 4) other sleep disorders (highlighting the role of OSA) are consistently associated with prevalent and incident markers of atherosclerosis^10–12^ but were not studied in the present investigation; 5) the applicability of the results to non-European populations deserve further investigations.

In conclusion, the study conducted by Xu et al.^8^ sheds light on the complexity of sleep and atherosclerosis in terms of appropriate characterization and how to estimate the risk of sleep disorders considering genetics and other traditional risk factors for atherosclerosis. As a potential cardiovascular risk factor, sleep research is still a ‘new kid on the block’ in Cardiology deserving further and appropriate investigations.^12^
